# Preparation of Primary Rat Hepatocyte Spheroids Utilizing the Liquid‐Overlay Technique

**DOI:** 10.1002/cptx.87

**Published:** 2019-09-13

**Authors:** Jonathan A. Kyffin, Christopher R. Cox, Joseph Leedale, Helen E. Colley, Craig Murdoch, Pratibha Mistry, Steven D. Webb, Parveen Sharma

**Affiliations:** ^1^ MRC Centre for Drug Safety Science, Department of Molecular and Clinical Pharmacology University of Liverpool Liverpool United Kingdom; ^2^ EPSRC Liverpool Centre for Mathematics in Healthcare, Department of Mathematical Sciences University of Liverpool Liverpool United Kingdom; ^3^ School of Clinical Dentistry, Claremont Crescent University of Sheffield Sheffield United Kingdom; ^4^ Syngenta Ltd. Jealott's Hill International Research Centre Bracknell Berkshire United Kingdom; ^5^ Department of Applied Mathematics Liverpool John Moores University Liverpool United Kingdom; ^6^ Current address: Department of Biological Sciences University of Chester Chester United Kingdom

**Keywords:** bile canaliculi, immunofluorescence, liquid‐overlay technique, liver spheroids, primary rat hepatocytes

## Abstract

Herein, we describe a protocol for the preparation and analysis of primary isolated rat hepatocytes in a 3D cell culture format described as spheroids. The hepatocyte cells spontaneously self‐aggregate into spheroids without the need for synthetic extracellular matrices or hydrogels. Primary rat hepatocytes (PRHs) are a readily available source of primary differentiated liver cells and therefore conserve many of the required liver‐specific functional markers, and elicit the natural in vivo phenotype when compared with common hepatic cells lines. We describe the liquid‐overlay technique which provides an ultra‐low attachment surface on which PRHs can be cultured as spheroids. © 2019 The Authors.

**Basic Protocol 1**: Preparation of agarose‐coated plates

**Basic Protocol 2**: Primary rat hepatocyte isolation procedure

**Basic Protocol 3**: Primary rat hepatocyte spheroid culture

**Basic Protocol 4**: Immunofluorescent analysis of PRH spheroids

## INTRODUCTION

Drug‐induced hepatotoxicity remains one of the leading causes of global acute liver failure, which can lead to patient hospitalization, discontinuation of essential and life‐preserving treatments, and the need for liver transplantation. It is also one of the leading causes of drug attrition during several stages in the drug development process. This is partially due to our incomplete understanding of drug metabolism and clearance in human systems that results from the unavailability of relevant and accessible translational in vitro liver models. Therefore, the development of improved in vitro platforms to assess liver toxicity after repeat‐dose exposure to xenobiotics is crucial to efficiently bring new compounds safely to market in a cost‐effective and timely manner.

Three‐dimensional (3D) culture techniques, such as supporting extracellular matrices (ECM), hydrogels, hanging‐drop cultures, and spheroids, have been shown to more closely recapitulate the in vivo microenvironment, allowing for extended viable culture periods compared to conventional two‐dimensional (2D) cell cultures (Godoy et al., 2013; Kyffin et al., 2018). The culturing of cells in a spheroid conformation increases the number of cell‐cell and cell‐ECM interactions, and these interactions mediate the behavioral and phenotypic characteristics of the cells, allowing for cultures to mimic the in vivo situation in a more representative way (Kyffin et al., 2019). There have been a number of published articles describing methods by which to produce spheroids, including the much utilized hanging‐drop method. A notable paper describing this culture platform was published by Messner and colleagues, and this method has now been fully commercialized (i.e., by InSphero) such that ‘ready‐made’ plates of spheroids can be purchased (Messner, Agarkova, Moritz, & Kelm, 2013). A significant hindrance to the widespread adoption of this commercial model and other similar platforms as early‐stage screening tools, both in research and in industry, is their considerable cost. Consequently, there has been a requirement for the development and implementation of simpler and cheaper alternatives.

Although other methods have been used to produce spheroids, such as rocked cultures and spinner flasks, a caveat of these approaches is the inability to control the resulting size of the spheroids, leading to limitations in precision when comparing results across studies. Moreover, the inability to control the size of spheroids leads to a more substantial problem whereby oxygen and other vital nutrients are unable to diffuse into the central regions of larger spheroids, and this can result in cellular necrosis due to hypoxia (Anada, Fukuda, Sai, & Suzuki, 2012). This article describes an efficient method to generate and culture primary rat hepatocyte (PRH) spheroids using the liquid‐overlay technique (LOT). Alternatives will also be given to allow choice of protocols based on time and financial availability. We also describe the hepatocyte isolation procedure in detail, as well as the main immunofluorescence protocol used to characterize the properties of the resultant PRH spheroids.

This article begins with the standard protocol for the production of agarose‐coated, ultra‐low attachment (ULA) plates (Basic Protocol 1), followed by the complete PRH isolation procedure using livers of male Wistar rats (Basic Protocol 2). We also describe the basic culture conditions required for the maintenance of the PRH spheroids for extended periods when implementing the LOT (Basic Protocol 3), and the specific immunofluorescence protocol for the staining and imaging of bile cannalicular‐like structures via P‐glycoprotein (P‐gp) immunostaining (Basic Protocol 4).

*NOTE*: All protocols using live animals must first be reviewed and approved by the local Animal Welfare and Ethical Review Body and must conform to governmental regulations regarding the care and use of laboratory animals. Additionally, these protocols are to be carried out in accordance with the principles of the Basel Declaration and recommendations of the ARRIVE guidelines issued by the NC3Rs. All work must be authorized by the Home Office under the Animals (Scientific Procedures) Act 1986 and the EU Directive.

*NOTE*: The procedures involving cell cultures are to be performed in a Class II biological safety cabinet, and everything entering the hood must be sprayed with a 70% alcohol solution. All solutions and equipment coming into contact with PRH cultures post‐isolation must be sterile, and aseptic technique must be implemented accordingly.

*NOTE*: All culture incubations should be performed in a humidified 37°C, 5% CO_2_ incubator unless specified otherwise.

## Basic Protocol 1 PREPARATION OF AGAROSE‐COATED PLATES

This protocol describes the necessary procedure for the preparation of agarose‐coated ULA plates required for PRH spheroid culture. The underlying principle for the production of spheroids using this methodology is that monodispersed cells are capable of reforming a 3D configuration via self‐reaggregation if adhesion to the substrate in which they are being cultured is prevented (Kelm et al., 2006). Furthermore, the prevalent theory of self‐assembly also suggests that, in the absence of external influences, cells will self‐organize into a spherical conformation (Napolitano, Chai, Dean, & Morgan, 2007).

### Materials

Agarose (high gelling temperature; Merck‐Sigma A7174)
Williams’ Medium E (basal medium; Merck‐Sigma W1878)

Digital scale, calibrated
25‐ml pipette (Thermo Fisher Scientific Sterilin 10693961) and pipette controller
250‐ml Pyrex glass medium bottle
Autoclave tape
Sterile reservoirs (STARLAB E2310‐1010)
8‐channel multichannel pipettor
200‐µl filter pipette tips (Elkay AER‐REF‐S96)
96‐well flat‐bottom sterile cell culture plate (Thermo Fisher Scientific 10212811)
Zip‐lock bags

### Production of liquid‐overlay plates

**Figure 1:**
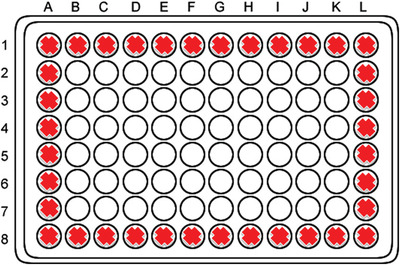
Spheroid culture plate layout. Red crosses indicate the wells that are not used for spheroid culture. All other wells are used to seed PRH for spheroid generation.

1 Weigh out 3 g of agarose using a calibrated digital scale.
2 Using a pipette controller and 25‐ml pipette, transfer 200 ml of basal Williams’ Medium E into a 250‐ml Pyrex medium bottle and add the agarose. Mix the solution by inverting the bottle. Note that the agarose will not dissolve until it is heated in the autoclave.
3 Loosely screw the lid onto the Pyrex medium bottle and secure the lid using autoclave tape. Autoclave this solution.
  3 g of agarose and 200 ml of basal medium will make enough agarose to coat approximately 16 plates.
4 After autoclaving has finished, ensure that the agarose is completely molten by microwaving prior to coating culture plates.
  Note that solution will be extremely hot, and care must be taken in handling.
5 In the sterile tissue culture hood, pour the autoclaved solution into sterile reservoirs and pipette 100 µl agarose solution (ensure that there are no air bubbles) into the inner 60 wells of a 96‐well flat bottom sterile cell culture plate using an 8‐channel pipettor.
  Note that the outer wells will not be used to culture spheroids due to a boundary evaporation effect. See Figure 1 for culture plate layout.

6 Allow agarose to set in the tissue culture hood for at least 30 min with the lids left off the 96‐well plates.
7 Replace the lids of the 96‐well plates and seal in plastic ziplock bags. Date the bags and ensure that the plates are stored inverted at 4°C.
  Plates can be stored for up to 4 weeks.
8 Leave culture plates at 4°C for at least 2 weeks to allow for appropriate hydration prior to cell seeding.
  Using plates within this 2‐week period can result in malformation of spheroids.
  If cost is not a limiting factor, premade ULA plates (Corning 96‐well Ultra Low Attachment; Corning Life Sciences; ThermoFisher Scientific 10023683) can be purchased and substituted for the agarose‐coated plates for PRH spheroid generation.

## Basic Protocol 2 PRIMARY RAT HEPATOCYTE ISOLATION PROCEDURE

This procedure describes the principal methodology for the isolation of hepatocytes from the whole liver of a young adult male Wistar rat, weighing between 175 and 200 g, and the subsequent purification steps for these parenchymal cells. This protocol is a modified version of the two‐step collagenase perfusion technique described originally by Seglen (1976). The procedure need not be conducted in an aseptic environment, as the culture of the PRHs is considered relatively “short‐term.” In order to achieve the highest number of healthy and viable hepatocytes during the isolation, well‐prepared, planned dissection and isolation are essential. This is dependent upon the use of fresh materials and solutions, usually prepared on the morning of the isolation procedure or the night before (e.g., for a Monday isolation, buffers may be prepared on the preceding Friday and stored at 4°C). Additionally, minimization of the time between anesthesia and the purification of isolated hepatocytes is vital. A single liver from a rat typically provides approximately 300 million hepatocytes (Cho, Berthiaume, Tilles, & Yarmush, 2008). A single rat liver isolation would, therefore, provide hepatocytes for numerous rounds of spheroid production and hundreds of potential cultures. As such, we suggest the implementation of tissue‐sharing schemes as endorsed by the NC3Rs; alternatively, cell suspensions may be cryopreserved and used for other studies at a later date (Stevenson, Morgan, McLellan, & Helen Grant, 2007).

### Materials

Wash buffer for hepatocyte isolation (see recipe), prewarmed to 47°C for a minimum of 30 min
Digestion buffer (see recipe), prewarmed to 47°C for a minimum of 30 min
Centrifugation buffer (see recipe), prewarmed to 47°C for a minimum of 30 min
Complete Williams’ Medium E (see recipe)
Collagenase A (from *Clostridium histolyticum*; Roche 11088793001)
DNase I (Merck‐Sigma D5025)
Trypsin inhibitor (type 1‐S from soybean; Merck‐Sigma 10109886001)
70% ethanol
Male Wistar rat (175‐200 g)
Sagatal (anesthetic; Rhone Merieux Ltd., Harlow, U.K.)
0.4% (w/v) trypan blue (Thermo Fisher Scientific 15250061)

5‐ml bijou bottles
50‐ml conical tubes (e.g., Corning Falcon)
Blue roll
Dissection tray
Tape
Cotton tie
Cotton swabs
Petri dish
Dissection tools including:
  Surgeon's scissors
  Cross‐action forceps
  Iris scissors
  Blunt forceps
125‐µm nylon gauze
250‐ml glass beaker
25‐G needle (VWR 613‐0902)
1‐ml syringe (Appleton Woods BD572)
18‐G inflow cannula (portal vein)
Ziplock bags
Perfusion apparatus including:
  Peristaltic pump
  Tubing
  Glass vial for bubble trap
Heated water bath at 37°C
0.5‐ml microcentrifuge tubes
Hemacytometer and coverslip
Inverted microscope

### Preparation for hepatocyte isolation and culture

Prepare wash buffer (for hepatocyte isolation), digestion buffer, and centrifugation buffer on the day before the isolation procedure.

### Pre‐surgery setup (morning of rat hepatocyte isolation)

1 Pre‐warm the wash buffer for hepatocyte isolation, digestion buffer, and centrifugation buffer at 47°C for a minimum of 30 min.
2 Pre‐warm the culture medium (complete Williams’ Medium E) at 37°C.
3 Pre‐cool the centrifuge at 4°C.
4 Weigh out into three separate 5‐ml bijou bottles:
  50 mg collagenase
  6.8 mg trypsin inhibitor
  20 mg DNase I.
  Store bijous on ice along with two 50‐ml conical tubes.
5 Set up culture hood for cell washing—spray hood down with 70% ethanol and put in necessary equipment.
6 Set up surgical tray for isolation, cotton swabs, blue roll, tape, cotton, petri dish, and dissection tools.
7 Cut nylon gauze squares ready for isolation (approximately 12 × 12 cm).
8 Clean 250‐ml beaker with distilled water, spray with 70% ethanol, and rinse again with distilled water.
9 Place 25‐G needle, 1‐ml syringe, and 18‐G cannula next to the dissection tray.
10 Prepare an additional ziplock bag.

### Set up for surgery

11 Prime the perfusion apparatus with the pre‐warmed wash buffer for hepatocyte isolation (∼50 ml; remove as much air as possible). Ensure that the bubble trap contains only buffer and no air bubbles. Ensure that the wash buffer is eluting from the syringe, and begin the procedure.
12 Secure the bubble trap in the clamp and arrange tubing through the pump teeth so that tubing is able to reach the water bath and easily reach the bubble trap and surgical area.
13 Adjust the perfusion flow rate to approximately 20 ml/min. Transfer the tubing to wash buffer for hepatocyte isolation.
  It is vital to adjust the flow rate here appropriately, as a too‐low flow rate can lead to incomplete perfusion and low hepatocyte yield, while a too‐high flow rate can compromise hepatocyte viability. For higher flow rates, a cotton tie is used to facilitate the retention of the cannula in the portal vein under flow.

### Prepare rat for surgery

14 Using a 25‐G needle fixed on a 1‐ml syringe, a dose of Sagatal (1 µl/g) is used to anaesthetize the rat. The fur at the site of perfusion is wiped with 70% ethanol to minimize any contamination of the hepatocytes.
15 After the rat is immobilized and the eyes stop blinking, the animal is transferred to the surgical tray which has been pre‐lined with blue roll, and the animal's limbs are secured to the tray with tape, ventral side up.
16 Ensure that the animal is insensate before proceeding by pinching firmly on the rear foot pads and watching for a reflex response. Wait for cessation of reflex responses before initiating surgical procedure.
  In general, this should take between 5 and 10 min.

### Perform surgery

17 A V‐shape incision is made through both the skin and muscle from the center of the lower abdomen to the rib cage. The guts are moved to the right to reveal the hepatic portal vein, and a cotton tie is placed around the vein.
18 The diaphragm is opened and the ribs are cut at either side and pinned back above each forepaw to reveal the thoracic cavity.
19 The hepatic portal vein is then cannulated with an 18‐G cannula, the inner needle is removed to leave the cannula in the vein, and the cotton tie is then tightened. At this point, the heart is removed to allow free flow of perfusion buffers.
20 Perfuse the liver with the wash buffer and start the timer for 9 min.
  Successful cannulation will be followed by a rapid blanching of the tissue, which will become a cream/yellow color.
  Detailed visual instructions on rat hepatocyte isolation can be found in Shen, Hillebrand, Wang, & Liu (2012).

### Digestion of the liver tissue

21 When the liver has been perfused for 7 min with wash buffer, add the collagenase A (see step 4; stored on ice) and trypsin inhibitor (see step 4; stored on ice) to the pre‐warmed digestion buffer (see step 1) and mix gently. Following perfusion with wash buffer, stop pump and transfer tube to perfuse with digestion buffer.
22 Perfuse with digestion buffer until the liver is clearly digested under the capsule (approximately 2 min).
23 While the liver is digesting, prepare the centrifugation buffer (for the release of the cells into the petri dish). Add 20 mg DNase I (see step 4; stored on ice) to 200 ml wash buffer for hepatocyte isolation.

### Isolation and purification of hepatocytes

24 At the end of the digestion, stop the perfusion and transfer the digested tissue to a plastic petri dish containing some of the centrifugation buffer made above in step 23 (wash buffer + DNase I).
25 Anchor the tissue and break the capsule with cross‐action forceps. Disturb the tissue gently with the forceps to release the cells into the centrifugation buffer.
26 Remove the residual tissue and filter the cell suspension through a 125‐µm nylon gauze, which has been pre‐wetted with the centrifugation buffer, into the sterile beaker (see step 8). Repeat until few cells are appearing in the buffer.
27 Split the filtered cell suspension into the two 50‐ml pre‐cooled conical tubes (see step 4) and allow cells to settle for approximately 10 min on ice.
28 Remove the supernatant and gently resuspend the cells in the centrifugation buffer. Spin tubes for 2 min at 50 × *g*, 4°C.
29 Remove the supernatant and resuspend in complete Williams’ Medium E (25 ml; see step 2).
30 Centrifuge tubes 2 min at 50 × *g*, 4°C.
31 Remove the supernatant and resuspend each pellet in complete Williams’ Medium E (25 ml).
32 Spin tubes 2 min at 50 × *g*, 4°C.
33 Remove the supernatant and pool cells and resuspend pellet in 40 to 50 ml of complete Williams’ Medium E.

### Cell counting and viability measurements

34 To a 0.5‐ml microcentrifuge tube, add 90 µl trypan blue and 10 µl of the cell suspension. Mix well.
35 Put 10 µl of the resulting suspension onto a hemacytometer. Count total cells and blue cells in four quadrants (Q).
36 Calculate the number of viable cells in the preparation:
  For each Q, calculate the number of viable cells:
Viable  cells  in  quadrant 1Q1= total  cells − blue  cells  dead  cells
  Then calculate the number of viable cells isolated:
Mean  number  of  viable  cells  per  quadrant Q¯=Q1+Q2+Q3+Q44
Viable  cells  per  ml =Q¯×10,000× dilution  factor
  where dilution factor = 10
Total  number  of  viable  cells  in  suspension = viable  cells  per  ml × total  volume  of  cell  suspension  in  ml
37 Viability must be ≥85% in order for cell culture to proceed successfully

## Basic Protocol 3 PRIMARY RAT HEPATOCYTE SPHEROID CULTURE

This protocol has been designed to generate homogenous 3D spheroids from freshly isolated PRHs. It provides instructions as to the spheroid culture conditions required for freshly isolated PRHs. To obtain the cells for 3D spheroids, rat hepatocytes are isolated from young male Wistar rats (Basic Protocol 2). Using LOT or ULA plates, PRH spheroids are generated from a specified initial cell seeding density, resulting in spheroids with a uniform size and reproducible phenotypic characteristics. This protocol is initiated directly after the isolation procedure as described in Basic Protocol 2.

### Materials

Freshly isolated PRHs (stored on ice)
Complete Williams’ Medium E (see recipe)

Pre‐prepared agarose‐coated 96‐well plates (see Basic Protocol 1; minimum of2 weeks at 4°C for appropriate plate hydration)
Sterile reservoirs (STARLAB E2310‐1010)
200‐µl filter tips (Elkay AER‐REF‐S96)
Multichannel pipettor
Sterile phosphate‐buffered saline (PBS), pH 7.2 (Thermo Fisher Scientific 20012019)
Centrifuge with rotor adapter for 96‐well plates

**Figure 2:**
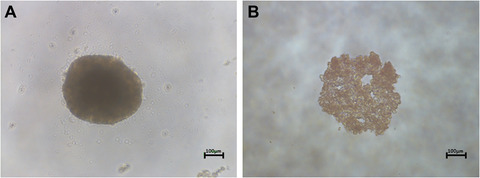
Morphology of spheroids produced using the liquid‐overlay technique (LOT). (**A**) A phase‐contrast image of a properly formed spheroid produced using 4000 PRH at day 3. (**B**) An example where a spheroid has failed to form correctly. Scale bar = 100 µm.

1 Agarose‐coated 96‐well plates are pre‐warmed in an incubator at 37°C for 30 min prior to cell seeding.
2 Agarose‐coated 96‐well plates are then left to air dry in laminar flow cabinet in order to remove condensation from plate lids.
  IMPORTANT NOTE: This is a vital step in the procedure, as condensation remaining in the lids considerably affects the ability to form spheroids.
3 Determine how many cells are required for each spheroid and dilute the cell solution to the correct concentration (note that each spheroid is cultured in 100 µl complete Williams’ Medium E). For example, for 2000 cells/spheroid, we require 2000 cells/well in 100 µl, i.e., a cell density of 20,000 cells/ml. Use the C_1_V_1_ = C_2_V_2_ rule to dilute the cells to the appropriate concentration, taking into account that we require 60 spheroids per plate:
  C_1_ = Cell density calculated above
  C_2_ = Cell density required (e.g., 20,000 cells/ml)
  V_2_ = Volume required (e.g., 6 ml per plate. Always make in excess—we suggest 7 ml per plate)
  V_1_ = Calculate the volume of cell suspension to add to volume required.
  The number of cells required for each spheroid is dependent on the individual experiment. However, past a certain size, a necrotic core will form. For further details, please refer to Kyfinn et al. (2019).
4 Add 100 µl of cell suspension to each of the inner 60 wells of the 96‐well agarose‐coated plate, using a sterile reservoir, multichannel pipettor, and 200‐µl filter tips.
5 Fill the outer 36 wells of the plate with sterile PBS.
6 After all plates have been seeded with cells, the plates are briefly pulse‐centrifuged—90 sec at 100 × *g*.
  This is required to ensure that all cells seeded go to the the bottom and center of the well. This will bring cells into close contact and increase the likelihood of spheroid formation.
7 Incubate for 72 hr undisturbed at 37°C with 5% CO_2_ until compact spherical structures are visible (ensure that the incubator is not opened and closed within the first 72 hr, as this will disturb the formation of the spheroids).
8 Change the medium twice weekly by removing 50 µl old medium and adding 50 µl new complete medium.
  For PRHs isolated on a Monday, medium changes are required on Thursday (day 3) and the following Monday (day 7). Cell seeding between 2000 and 5000 cells results in the formation of spheroids of approximately 200 to 500 µm in diameter, which are visible by eye. See Figure 2 for examples of a properly formed PRH spheroid typified by a regular and smooth encapsulating membrane around a single spheroid (Fig. 2A). Figure 2B exemplifies the failed formation of spheroids, where the cells have collected in the center of the well but have not resulted in the formation of a single 3D spheroid.

## Basic Protocol 4 IMMUNOFLUORESCENT ANALYSIS OF PRIMARY RAT HEPATOCYTE SPHEROIDS

In our complex spheroid model, the analysis of cellular morphology and location of substructures is facilitated via the implementation of immunofluorescent staining. Immunofluorescence uses a primary antibody against cell‐specific markers and a secondary antibody linked to a fluorophore, a fluorescent compound that can emit a detectable light signal upon excitation with specific wavelengths of light. We implemented this protocol to analyze and visualize the formation of secondary structures such as bile‐canaliculi‐like structures that form throughout the PRH spheroid model, and to ensure that in vivo‐like cellular polarization of hepatocytes is achieved.

### Materials

Spheroids in 96‐well culture plate (Basic Protocol 3)
Phosphate‐buffered saline (PBS), pH 7.2 (Thermo Fisher Scientific 20012019)
4% paraformaldehyde pH 7.2 (Merck‐Sigma P6148)
Permeabilization buffer (see recipe)
Block buffer (see recipe)
Anti‐P‐gp primary antibody [EPR10364‐57] (Abcam ab170904)
Alexa Fluor 568 donkey anti‐mouse secondary antibody (Abcam ab175472)
Immunofluorescence wash buffer I and II (see recipe)
Hoechst 33342 stain (Abcam ab228551)
Phalloidin 680 stain (Thermo Fisher Scientific A22286)
Prolong Gold Antifade Mountant (Thermo Fisher Scientific P36934)
Clear nail polish

96‐well microplate (Thermo Fisher Scientific 10212811)
20‐µl filter tips (Elkay AER‐OREF‐S96)
200‐µl filter tips (Elkay AER‐REF‐S96)
1000‐µl filter tips (Elkay AER‐STE1‐A18)
P20 Gilson repeat pipettor
P200 Gilson repeat pipettor
P1000 Gilson repeat pipettor
Aluminum foil
Microscope slide, Superfrost, 76 mm × 26 mm (Thermo Fisher Scientific 10149870)
Cover slips 22 mm × 50 mm (Fisher Scientific 12373128)
Kimwipes
Fluorescence microscope

### Day 1: Fixation and permeabilization of spheroids

1 In the class II biological safety cabinet, spheroids to be stained for immunofluorescence are transferred from the cell culture plate into a fresh 96‐well microplate (not agarose‐coated) using a P200 repeat pipettor with a 200‐µl tip.
  These PRH spheroids produce high levels of extracellular matrix structures throughout the microtissue, and therefore transferring spheroids from one plate to another does not disrupt the structure.
2 Remove the new plate containing the spheroids from the hood for staining.
  These are going to be fixed, and therefore no longer require aseptic handling.
3 Remove medium using a 200‐µl repeat pipettor and 200‐µl filter tips. Wash the spheroids three times, each time by adding 100 µl PBS and then removing the PBS.
  100‐µl volume is used per well for all subsequent steps including antibody incubations.
4 Fix spheroids with 4% paraformaldehyde for 1 hr at 4°C.
5 Remove the paraformaldehyde using a 200‐µl repeat pipettor and a 200‐µl filter tip and discard in a chemical waste bin. Wash the spheroids by adding 100 µl of immunofluorescence wash buffer I and then removing it with a 200‐µl repeat pipettor and a 200‐µl filter tip. Repeat this three times.
  After this step, spheroids can be resuspended in PBS, pH 7.2, and left for ∼2 weeks at 4°C in a sealed plate.
  Cells are then permeabilized to allow the penetration of antibodies inside the cell.
6 100 µl of permeabilization buffer is added to each well and the spheroids kept at 4°C overnight.

### Day 2: Blocking and incubation with primary antibody

Nonspecific binding is blocked by the addition of bovine serum albumin (BSA). In this study, we monitor hepatocellular polarization and the presence of bile canalicular by the fluorescent localization of P‐gp, which is present at the bile canalicular membrane.

7 Remove permeabilization buffer and add 100 µl block buffer for 2 hr at room temperature.
8 Dilute Anti‐P‐gp primary antibody 1:50 in block buffer.
9 Remove the block buffer and add 100 µl of the primary antibody dilution from step 8. Cover the plate with foil and incubate spheroids overnight at 4°C.
  As a negative control, incubate additional spheroids without the primary antibody.

### Day 3: Secondary antibody and stains

In order to detect immunofluorescence, secondary antibodies are conjugated with fluorescent tags that are excited at specific wavelengths.

10 Spheroids are washed with immunofluorescence wash buffer II three times, each time for 1 hr at room temperature.
11 Dilute Alexa Fluor 488 donkey anti–mouse secondary antibody (1:1000), Hoechst 33342 stain (1:5000), and phalloidin 680 (1:250) together in block buffer.
12 Remove wash buffer and add 100 µl/well of the secondary antibody/stain mixture prepared in step 11. Note that secondary antibodies are light sensitive and, at this stage, exposure to light should be minimized. Cover the plate with foil and incubate the spheroids overnight at 4°C (without rocking).

### Day 4: Mount cells for visualization

In order to maintain cells for immunofluorescent visualization, the spheroids are mounted and maintained in Prolong Gold fixative. Throughout this stage of the protocol, exposure to light should be minimized.

**Figure 3:**
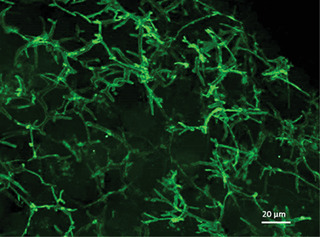
P‐gp staining of bile canalicular formation throughout spheroids. 3000‐cell spheroids were cultured on liquid‐overlay plates for 3 days, fixed, and stained with P‐gp (green). A maximum intensity projection image was taken at 40× magnification. Scale bar = 20 µm.

13 Spheroids are washed with 100 µl wash buffer I for 1 hr at room temperature in the dark.
14 Transfer spheroid in a small amount of buffer to a Superfrost microscope slide using a P1000 repeat pipettor and 1000‐µl filter tip (this ensures that spheroids remain undamaged). Remove excess liquid from the slide using a pipette and Kimwipe.
15 Add approximately 10 µl of Prolong Gold Antifade Mountant using a P20 repeat pipettor and 20‐µl filter tip to cover the spheroid, and gently place coverslip on top.
  Be careful to avoid creating bubbles in the mountant, as this will interfere with imaging analysis. Try to avoid applying too much pressure with the coverslip, as this will distort the spheroid.
16 Use nail polish to seal the coverslip on the slide and let it dry in the dark for approximately 30 min.
17 Image analysis can be carried out as soon as slides have dried, or slides can be stored in a slide box at 4°C.
  Cells will maintain fluorescent signals over a few months (see Fig. 3 for an example of P‐gp‐stained PRH spheroid and the type of results that should be expected from the immunofluorescent analysis described in this protocol).

## REAGENTS AND SOLUTIONS

### Centrifugation buffer (200 ml)

200 ml wash buffer (see recipe)
20 mg DNase I ((Merck‐Sigma D5025; add as described in Basic Protocol 1)
Prepare fresh for each use

### Complete William's medium E

William's medium E basal medium (Merck‐Sigma W1878) containing:
10% heat‐inactivated FBS (Thermo Fisher Scientific A3840401)
1% penicillin‐streptomycin (100×; Thermo Fisher Scientific 15140122) giving a final concentration of 100 units/ml penicillin and 0.1 mg/ml streptomycin
2 mM L‐glutamine (Thermo Fisher Scientific 25030032)
10 µg/ml insulin, 5.5 µg/ml transferrin, 6.7 ng/ml selenium (add as insulin/transferrin/selenium solution; Thermo Fisher Scientific 41400045)
100 nM dexamethasone (Merck‐Sigma D4902)
Store up to 1 month at 4°C

### Digestion buffer (100 ml)

99.5 ml of wash buffer (see recipe)
0.5 ml of 1.325 M CaCl_2_ solution consisting of 73.5 mg calcium chloride dihydrate (CaCl_2_·2H_2_O; Merck‐Sigma C7902) dissolved in 0.5 ml deionized water
Prepare fresh for each use

### Immunofluorescence block buffer

Immunofluorescence permeabilization buffer (see recipe) plus 3% BSA. Store up to 1 week at 4°C.

### Immunofluorescence permeabilization buffer

Phosphate‐buffered saline (PBS; Thermo Fisher Scientific 20012019) containing:
  0.5% (v/v) Tween 20
  0.2% (v/v) Triton X‐100
  Store up to 1 week at 4°C

### Immunofluorescence wash buffer I

1× phosphate‐buffered saline (PBS; Thermo Fisher Scientific 20012019).

### Immunofluorescence wash buffer II

Phosphate‐buffered saline (PBS; Thermo Fisher Scientific 20012019) containing:
  0.1% Tween
  0.2% Triton‐X100
  Store up to 1 week at 4°C

### Wash buffer for hepatocyte isolation (1 L)

100 ml 10× Hanks balanced salt solution (HBSS; Thermo Fisher Scientific 14185045)
1.38 g HEPES (Merck‐Sigma H4034)
900 mM sodium bicarbonate (NaHCO_3_) (Merck‐Sigma S5761; 378.05 mg NaHCO_3_ in 5 ml of deionized water)
895 ml deionized water
Prepare fresh for each use

## COMMENTARY

### Background Information

Methods for culturing rat hepatocytes in 2D have been established for a number of years, but to our knowledge, no definitive studies have described conditions showing the implementation of LOT and agarose‐coated ULA plates for the formation of primary rat liver spheroids. In more recent years, 3D cultures of hepatocytes and hepatic‐derived cell have become increasingly utilized for hepatotoxicity investigations due to the rapid development of more amenable, cost‐effective, and uncomplicated culture methodologies. One of the major limitations to the mainstream adoption of commercially available 3D liver models is their cost; as such, the need for more cost‐effective approaches has been a considerable driver in development of bioengineered liver culture models. Using the protocols in this article, isolated PRHs can be cultured for at least 30 days in agarose‐coated plates, which provide a ULA surface for spheroid formation. These plates cost a fraction of the price of their commercially available counterparts. Thus, this model provides an adequate experimental platform to carry out early‐stage hepatotoxicity studies, related to human risk potential, for novel xenobiotic development across industry sectors. More specifically, due to the formation of functional bile‐canalicular structures, the described PRH spheroids may provide further insight into the mechanisms of hepatobiliary transport–associated manifestations of toxicity.

One of the key advantages that 3D liver cell cultures have over their 2D counterparts is that the complexity of the native microenvironment is conserved over extended periods. The recent progress in 3D in vitro liver spheroid models may lead to an improved ability to predict hepatotoxicity of novel compounds, owing to the establishment of a more physiologically relevant environment as seen in the in vivo liver (Andersson, 2017). The literature has reported that the re‐establishment of cellular polarization is a vital process that facilitates the maintenance of gene expression and hepatocyte‐specific function (Dunn, Tompkins, & Yarmush, 1991). 2D liver cultures have a limited capability for establishing polarized cellular conformations that display the multiple apical and basolateral membranes as seen in vivo. Therefore, it is crucial that novel and alternative in vitro liver models have the ability to restore this hallmark of liver physiology.

**Figure 4:**
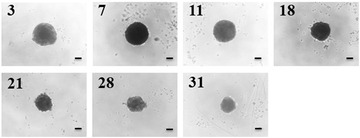
Compaction of 2000‐cell spheroids over 31 days of culture. Phase‐contrast images of spheroids were taken at days 3, 7, 11, 18, 21, 28, and 31 of the culture period. Scale bar = 100 µm.

Researchers have reported numerous 3D liver models that enable the establishment of cellular polarization, including hydrogel‐ and scaffold‐based technologies, as well as liver spheroids (Bell et al., 2016; Fang & Eglen, 2017; Gaskell et al., 2016; Knight, Murray, Carnachan, & Przyborski, 2011; Kyffin et al., 2019; Lee et al., 2015; Napolitano et al., 2007; van Zijl & Mikulits, 2010). The LOT method to culture PRH spheroids has a number of advantages over other methods for spheroid generation. Primarily, the size of the resulting spheroids is simply manipulated by changing the initial cell seeding density in the 100 µl of medium within the culture well. Secondly, this method of spheroid generation does not require any specialized and expensive equipment for experimental setup, and a single isolated liver from one animal provides a vast amount of cells to cover multiple experimental repeats. The LOT provides a culture platform where PRHs rapidly aggregate into a single central spheroid at the base of the agarose‐coated culture well, 72 hr post seeding. Compaction of the spheroid occurs over the duration of the culture time due to upregulation and expression of ECM components, resulting in a spheroid composed of polarized cuboidal hepatocytes embedded in matrix (see Fig. 4 for phase‐contrast microscopy images of compacting 2000‐cell spheroids over 31 days of culture).

### Critical Parameters and Troubleshooting

The viability of PRHs after the isolation procedure is vitally important for the appropriate culture of viable spheroids and for subsequent spheroid analysis. One of the critical considerations for the culture of PRH spheroids is ensuring that culture plates have been pre‐warmed and that once culture plates have been transferred into the culture hood for cell seeding, all of the condensation on the plates and lids has completely evaporated (pre‐warming and condensation evaporation within the plates takes approximately 45 to 60 min). The culturing of PRH spheroids utilizing the LOT is predominantly influenced by factors such as uninterrupted culture post‐seeding, adequate airflow in the incubator, and ensuring that the reagents are used at the same temperature, e.g., the complete Williams’ Medium E. Additionally, the maintenance of these critical parameters will ensure virtually complete aggregation and compaction of the PRHs into a single spheroid after 72 hr of culture. For proper formation of PRH spheroids by the LOT, isolated rat hepatocytes should be of high viability (>85%) and suspended at cell concentrations of ≥2000 cell per well. When seeding cells into the culture plates, careful mixing of the cell suspension in the sterile reservoirs before pipetting ensures a homogenous suspension and that equal and uniform‐sized spheroids will be produced. Furthermore, care must be taken when carrying out medium changes to ensure that spheroids are not damaged or lost. For immunofluorescence analysis, extreme care must be taken when mounting processed spheroids so they are not damaged or flattened and distorted. There have been numerous papers discussing potential limitations of spheroids, and, in particular, the incidence of necrosis due to hypoxia has been a key issue of these broader discussions. Although we have not included additional experimental and collaborating mathematical analysis in this manuscript related to our investigations of oxygen utilization, we can refer readers to Kyffin (2018) where we describe optimized operating conditions for this experimental spheroid model to recapitulate oxygen gradients as seen in the liver sinusoid in vivo. Furthermore, Chapters 3 and 4 in Kyffin (2018) describe model‐specific experimental and in silico modeling for oxygen utilization as well as xenobiotic distribution and cellular transport routes/bioavailability. In summary, we show that our specific PRH spheroid model remains devoid of necrosis for the entirety of the standard 31‐day culture period, and that by fine‐tuning external oxygen concentrations (i.e., oxygen in the incubators), in vivo sinusoidal oxygen tensions can be replicated.

### Understanding Results

The generation of PRH spheroids using the LOT cultured in agarose plates is expected to produce spheroids approximately 200 to 500 µm in diameter in each well when suspended in 100 µl of culture medium. This will depend on the initial cell seeding density chosen (our previous work has analyzed spheroids at 2000‐ 5000‐cell initial seeding densities; Kyffin et al., 2019). Approximately 90% of the initial amount of cells seeded are incorporated in the spheroid culture after 72 hr of culture in agarose plates. Spheroid cultures will reduce in size/diameter over the duration of the culture period due to the upregulation of ECM and cytoskeletal components, resulting in spheroidal compaction. This has been observed and well documented previously (Bell et al., 2016; Lin, Chou, Chien, & Chang, 2006). Immunofluorescent analysis with P‐gp should result in the staining of fine, bile‐canaliculi structures throughout the PRH spheroids. These structures are extensive and are expected to be approximately 0.5 to 2.5 µm.

### Time Considerations

The amount of time required for rat hepatocyte isolation was discussed in Basic Protocol 2, with this procedure generally taking up to 1 hr. Using this modified two‐step collagenase perfusion method, 3 × 10^8^ hepatocytes per rat liver can be obtained, with an average viability ≥85%. Based on the cell seeding density described in Basic Protocol 3, usually one experimental setup requires seven plates of spheroids per seeding density (2000, 3000, 4000, and 5000 cells per well in 60 wells). Therefore, one experimental setup requires approximately 5.88 × 10^6^ hepatocytes for all of our chosen cell‐seeding densities. Cells are then cultured using the LOT, and the culture medium changed twice weekly. The time required to change the medium depends on the number of plates. Generally, it takes approximately 1.5 hr to change the medium in 28 plates using a multichannel pipettor. This may take longer, as it is vital that spheroids not be removed/lost from the well during the changing of culture medium. The time required to complete the immunofluorescent staining protocol described in Basic Protocol 4 is approximately 4 days due to the overnight incubations required for primary and secondary antibodies.

## References

[cptx87-bib-0001] Anada, T. , Fukuda, J. , Sai, Y. , & Suzuki, O. (2012). An oxygen‐permeable spheroid culture system for the prevention of central hypoxia and necrosis of spheroids. Biomaterials, 33(33), 8430–8441. doi: 10.1016/j.biomaterials.2012.08.040. 22940219

[cptx87-bib-0002] Andersson, T. B. (2017). Evolution of novel 3D culture systems for studies of human liver function and assessments of the hepatotoxicity of drugs and drug candidates. Basic & Clinical Pharmacology & Toxicology, 121(4), 234–238. doi: 10.1111/bcpt.12804. 28470941

[cptx87-bib-0003] Bell, C. C. , Hendriks, D. F. , Moro, S. M. , Ellis, E. , Walsh, J. , Renblom, A. , … Ingelman‐Sundberg, M. (2016). Characterization of primary human hepatocyte spheroids as a model system for drug‐induced liver injury, liver function and disease. Scientific Reports, 6, 25187. doi: 10.1038/srep25187. 27143246 PMC4855186

[cptx87-bib-0004] Cho, C. H. , Berthiaume, F. , Tilles, A. W. , & Yarmush, M. L. (2008). A new technique for primary hepatocyte expansion in vitro. Biotechnology and Bioengineering, 101(2), 345–356. doi: 10.1002/bit.21911. 18465801 PMC4487520

[cptx87-bib-0005] Dunn, J. C. , Tompkins, R. G. , & Yarmush, M. L. (1991). Long‐term in vitro function of adult hepatocytes in a collagen sandwich configuration. Biotechnology Progress, 7(3), 237–245. doi: 10.1021/bp00009a007. 1367596

[cptx87-bib-0006] Fang, Y. , & Eglen, R. M. (2017). Three‐dimensional cell cultures in drug discovery and development. SLAS Discovery, 22(5), 456–472. doi: 10.1177/1087057117696795. 28520521 PMC5448717

[cptx87-bib-0007] Gaskell, H. , Sharma, P. , Colley, H. E. , Murdoch, C. , Williams, D. P. , & Webb, S. D. (2016). Characterization of a functional C3A liver spheroid model. Toxicology Research, 5(4), 1053–1065. doi: 10.1039/C6TX00101G. 27746894 PMC5047049

[cptx87-bib-0008] Godoy, P. , Hewitt, N. J. , Albrecht, U. , Andersen, M. E. , Ansari, N. , Bhattacharya, S. , … Hengstler, J. G. (2013). Recent advances in 2D and 3D in vitro systems using primary hepatocytes, alternative hepatocyte sources, and non‐parenchymal liver cells, and their use in investigating mechanisms of hepatotoxicity, cell signaling and ADME. Archives of Toxicology, 87(8), 1315–1530. doi: 10.1007/s00204-013-1078-5. 23974980 PMC3753504

[cptx87-bib-0009] Kelm, J. M. , Djonov, V. , Ittner, L. M. , Fluri, D. , Born, W. , Hoerstrup, S. P. , & Fussenegger, M. (2006). Design of custom‐shaped vascularized tissues using microtissue spheroids as minimal building units. Tissue Engineering, 12(8), 2151–2160. doi: 10.1089/ten.2006.12.2151. 16968156

[cptx87-bib-0010] Knight, E. , Murray, B. , Carnachan, R. , & Przyborski, S. (2011). Alvetex®: Polystyrene scaffold technology for routine three dimensional cell culture. In J. W. Haycock (Ed.), 3D cell culture: Methods and protocols (pp. 323–340). Totowa, NJ: Humana Press. 10.1007/978-1-60761-984-0_20 21042981

[cptx87-bib-0011] Kyffin, J. A. (2018). Establishing species‐specific 3D liver microtissues for repeat dose toxicology and advancing in vitro to in vivo translation through computational modelling (Doctoral thesis, Liverpool John Moores University, North England, UK).

[cptx87-bib-0012] Kyffin, J. A. , Sharma, P. , Leedale, J. , Colley, H. E. , Murdoch, C. , Harding, A. L. , … Webb, S. D. (2019). Characterisation of a functional rat hepatocyte spheroid model. Toxicology in Vitro, 55, 160–172. doi: 10.1016/j.tiv.2018.12.014. 30578835 PMC6361770

[cptx87-bib-0013] Kyffin, J. A. , Sharma, P. , Leedale, J. , Colley, H. E. , Murdoch, C. , Mistry, P. , & Webb, S. D. (2018). Impact of cell types and culture methods on the functionality of in vitro liver systems – A review of cell systems for hepatotoxicity assessment. Toxicology in Vitro, 48C, 262–275. doi: 10.1016/j.tiv.2018.01.023. 29408671

[cptx87-bib-0014] Lee, B. H. , Kim, M. H. , Lee, J. H. , Seliktar, D. , Cho, N. J. , & Tan, L. P. (2015). Modulation of Huh7.5 spheroid formation and functionality using modified PEG‐based hydrogels of different stiffness. PLos One, 10(2), e0118123. doi: 10.1371/journal.pone.0118123. 25692976 PMC4333219

[cptx87-bib-0015] Lin, R. Z. , Chou, L. F. , Chien, C. C. , & Chang, H. Y. (2006). Dynamic analysis of hepatoma spheroid formation: Roles of E‐cadherin and beta1‐integrin. Cell and Tissue Research, 324(3), 411–422. doi: 10.1007/s00441-005-0148-2. 16489443

[cptx87-bib-0016] Messner, S. , Agarkova, I. , Moritz, W. , & Kelm, J. M. (2013). Multi‐cell type human liver microtissues for hepatotoxicity testing. Archives of Toxicology, 87(1), 209–213. doi: 10.1007/s00204-012-0968-2. 23143619 PMC3535351

[cptx87-bib-0017] Napolitano, A. P. , Chai, P. , Dean, D. M. , & Morgan, J. R. (2007). Dynamics of the self‐assembly of complex cellular aggregates on micromolded nonadhesive hydrogels. Tissue Engineering, 13(8), 2087–2094. doi: 10.1089/ten.2006.0190. 17518713

[cptx87-bib-0018] Seglen, P. O. (1976). Preparation of isolated rat liver cells. Methods in Cell Biology, 13, 29–83. 177845 10.1016/s0091-679x(08)61797-5

[cptx87-bib-0019] Shen, L. , Hillebrand, A. , Wang, D. Q. H. , & Liu, M. (2012). Isolation and primary culture of rat hepatic cells. Journal of Visualized Experiments, 64, e3917. doi: 10.3791/3917. PMC3471302 22781923

[cptx87-bib-0020] Stevenson, D. J. , Morgan, C. , McLellan, L. I. , & Helen Grant, M. (2007). Reduced glutathione levels and expression of the enzymes of glutathione synthesis in cryopreserved hepatocyte monolayer cultures. Toxicology in Vitro, 21(3), 527–532. doi: 10.1016/j.tiv.2006.11.005. 17196364

[cptx87-bib-0021] van Zijl, F. , & Mikulits, W. (2010). Hepatospheres: Three dimensional cell cultures resemble physiological conditions of the liver. World Journal of Hepatology, 2(1), 1–7. doi: 10.4254/wjh.v2.i1.1. 21160950 PMC2998947

